# Quackery

**Published:** 1856-04

**Authors:** J. W. Salisbury

**Affiliations:** Russelville, O.


### QUACKERY.

BY J. W. SALISBURY, M. D.

The word *Quack*, is usually employed to designate a person, who pretends to knowledge, which he does not possess; and professes to cure diseases, of the nature of which, he is entirely ignorant.

*Quackery* may properly be considered under three heads, viz., *Mountebankery*, *Empiricism* and *Charlatanism;* although the three are frequently united, and practised by the same individual.

A *Mountebank*, (from *Montare* and *Banco*, to mount a bench,) as originally used, signifies a person who mounts a bench or stage in public places, and, boasting of his skill in curing diseases, vends medicines which he pretends are infallible remedies.

Every day we see the operation of this class of Quacks. We are hailed in the street, we are accosted on the boat, we are met in the market, and solicited to buy their nostrums. Almost every newspaper we take up is *polluted* with brilliant deceitful advertisements, in praise of a *salve* that is equally efficacious in *curing* a *corn*, or *killing* a *cancer;* a world-renowned *balsam* that will speedily cure a *cold*, a *cough*, or a *confirmed consumption*, a *liniment* that will chase a *pain*, be it in *tooth* or *toe*, in the *hand* or the *heart*. Handbills are posted at almost every street-corner and alley, proclaiming the miraculous cures of this or that nostrum; but never even telling of a *single failure*, of its multiplied thousands. Their motto is 'NEVER FAILS.' !!!

Now one would think that a nostrum that is recommended as a certain, safe and speedy cure, for almost an endless number of diseases, would, in an enlightened community, find but few to try its virtues; but alas! men will be deceived. And it does often seem, that the more unreasonable assertions are, the more readily they are gulped down for truth.

If a medicine is advertised, "*never fails*," or "*no cure, no pay*," many will believe in its efficacy, and use it either for the sting of a bee, or for the cholera; and if it *fails* to *kill* in the first case, they readily conclude that it is *impossible* for it to *fail* to *cure* in the second.

Let the community once use their common sense in the matter, and patent medicines, with their humbugging originators will soon dwindle into obscurity.

A mechanic, in building a house must take timber piece by piece, and carefully prepare each for a particular place. He cannot expect to build a house by throwing them together at random. Neither can he expect a certain piece to answer equally well for a rafter or a sleeper.

So it is in the *practice of medicine.* Each dangerous symptom as they appear one after another, must be carefully watched and counteracted. In a disease we will perhaps one day find a *fever* that must be *controlled*, while the next day will reveal an *inflammation* that must be *checked;* while again the next we will be called upon to *stimulate* the sinking powers of nature in battling against the disease. Now what man with common sense, would pretend that the same course of treatment would be proper from the beginning to the end of such a case? Yet those who use a patent medicine for a disease, must necessarily, use it in the various stages of the disease. So we see that those, who are *duped* by these *mountebank preparations*, must inevitably risk the losing of their *health* and *lives;* particularly if the nostrum they employ *happens* to *possess any strength.*

But if it is a matter of surprise that the *common people*, should be thus deceived; how shall we express our astonishment, that even *learned*, *influential ministers of the Gospel* should give the influence of their *semi-holy names* to help to *humbug* their fellow-men. It does seem, to me at least, that the *minister of the Gospel of Christ*, who *professes* to be proclaiming the mandates of Heaven to a fallen race, and at the same time aiding impostors in deceiving their fellow-men, are committing a moral wrong, for which a *long life-time* of their professional labors, *without remuneration*, could not atone.

But the worst part is not yet told; for even *physicians* occasionally recommend a nostrum of which they know not the first ingredient. The *mechanic* may have excuse, the

*minister* even, may find excuse, but where under the sun can the *physician* try to find excuse, for recommending a nostrum professing to cure almost any and every disease in both man and beast. I would rejoice if I could say no *regular physician* has thus degraded himself. But alas! some even who claim to belong to the *regular profession*, have *bowed the knee to Moloch, and assisted insatiable, unconscionable vampires* in deceiving the community.

An *Empiric* (from the Greek, *en* and *peira*, "a trial,") signifies a man who enters upon the practice of medicine, without a regular professional education, or without otherwise qualifying himself for the station, he desires to occupy. The lives of human beings certainly are not so unimportant, that they should be thus *jeopardised* by the *unscientific hap-hazard experiments* of the *illiterate empiric.* Experiments even in medicine are necessary; and a man may experiment without being an empiric. But if a physician does not use the means within his reach to *inform himself*, he is an *empiric.* True, he may *accidentally* stumble upon a plan of treatment by which his patient is cured; or the "*vis medicatrix naturæ*" may perform the cure, despite his doses ill-applied.

And how often are the apparent cures of such empirics heralded over the land as *something marvelous*, being received with greater surprise the further they are proclaimed; while the honest, faithful, well-educated physician is *daily* performing cures *ten-fold greater*, of which nothing is heard or said a few miles distant. The fact is, it is only what is expected of a well-informed physician, while even an *accidental* cure by an ignorant empiric *is marvelous.*

A *Charlatan*, (from the Italian *Ciarlare*, "to talk much,") originally signified one who went from place to place to sell any medicine to which he ascribed wonderful sanative qualities: but the word is now used to denote one who boasts much of himself, and makes unwarrantable pretensions to skill. The members of the medical profession ought to labor together, as a band of brothers, for the welfare of their fellow-men. But alas! they are too often *warring* among themselves, each boasting what he *can do* and *has done*, while he is defaming the success of his brother.

Now the *true physician* is willing to spread before the world his discoveries and improvements in the art of medicine; in order, if possible, that the whole afflicted race of human beings may thereby be benefited. Their philanthropy would not permit them to circumscribe the benefits of their discoveries to the bounds of their own very limited practice.

But the Charlatan strives to keep every thing he knows to himself, and labors to convince the community that he knows a *little more* than *everybody else together.*

It is true we may frequently do *honor* to ourselves, in controlling diseases, but we should let *others* praise us. We should let our *works* and not our words establish our character.

A man that is *continually boasting* of his skill and uniform certainty in curing those diseases, which the body of the medical profession are *failing* to cure, should be looked upon with suspicion, and *denied* the *patronage* of an unsuspicious public.

Now if the above be true, there is work for us to do. 1st. A true statement of *facts* should be *published* and liberally spread before the whole community. Being enlightened they would be less often deceived. They would also join us in the work of reformation.

2d. We should labor to have a law enacted, *prohibiting* the sale of *secret patent nostrums.*

3d. A law should be enacted *prohibiting* the man who is *not qualified*, from *practising medicine.*

4th. *The physician* who claims *secrets* in *medicine* which he *refuses* to make known, should be treated by the profession as a "*Cheat* and a *Humbug*," a "whited sepulchre, filled with dead men's bones."

*Russelville, O.*, Feb. 10th, 1856.

